# Potential Role of Serum Cytokines and Chemokines as Biomarkers of Injury Severity and Functional Outcomes Following Pediatric Traumatic Brain Injury

**DOI:** 10.3390/cells15010019

**Published:** 2025-12-22

**Authors:** Kathryn Swaby, Alexander J. Skirvin, Natalie Machado, Maria Mateo Chavez, Julia Alexis Bernal, Ana Fuentes, Charlene P. Pringle, Kourtney Guthrie, Jennifer Coto, Rajderkar Dhanashree, Joslyn Gober, Paula Karina Perez, Juan P. Solano, Heather J. McCrea, Ricardo Loor-Torres, Joyce Kaufman, Ayham Alkhachroum, Kristine H. O’Phelan, Firas Kobeissy, Robert W. Keane, Kevin K. Wang, W. Dalton Dietrich, Juan Pablo de Rivero Vaccari, Jennifer C. Munoz Pareja

**Affiliations:** 1 Division of Pediatric Critical Care, Department of Pediatrics, University of Miami Miller School of Medicine, Miami, FL 33136, USA; 2 University of Miami Miller School of Medicine, Miami, FL 33136, USA; 3 Department of Pediatrics, University of Miami Volunteer, Miami, FL 33136, USA; 4 Knowledge and Evaluation Research Unit, Mayo Clinic, Rochester, MN 55905, USA; 5 Universidad Anáhuac, Huixquilucan 52786, Mexico; 6 Department of Pediatric Critical Care, University of Florida College of Medicine, Gainesville, FL 32610, USA; 7 Department of Pediatrics, University of Miami Concussion Program, University of Miami Miller School of Medicine, Miami, FL 33136, USA; 8 Division of Pediatric Radiology, Department of Radiology, University of Florida College of Medicine, Gainesville, FL 32608, USA; 9 Department of Pediatric Rehabilitation and Pediatrics, University of Miami Miller School of Medicine, Miami, FL 33136, USA; 10 Neurocritical Care, Department of Neurology, University of Miami Miller School of Medicine, Miami, FL 33136, USA; 11 Department of Neurological Surgery and Pediatrics, University of Miami Miller School of Medicine/Jackson Health System, Miami, FL 33136, USA; 12 Department of Surgery, University of Miami Miller School of Medicine, Miami, FL 33136, USA; 13 Department of Neurobiology, Center for Neurotrauma, Multiomics & Biomarkers (CNMB), Morehouse University, School of Medicine, Atlanta, GA 30310, USA; 14 Department of Neurological Surgery, Physiology and Biophysics and the Miami Project to Cure Paralysis, University of Miami Miller School of Medicine, Miami, FL 33136, USA

**Keywords:** pediatric traumatic brain injury, biomarkers, cytokines, chemokines, neuroinflammation

## Abstract

**Highlights:**

**What are the main findings?**

- Cytokines may be beneficial as biomarkers of pediatric TBI severity and prognosis

**What are the implications of the main findings?**

- Additional studies may be beneficial in delineating the role of cytokines in assessing pediatric TBI.

**Abstract:**

Traumatic brain injury (TBI) is one of the leading causes of death and neurological disability worldwide. The search for biomarkers that indicate TBI severity and prognosis with greater accuracy is ongoing. This study aimed to evaluate the significance of several neuroinflammatory cytokines and chemokines, assessing their potential as biomarkers in pediatric TBI (pTBI). This was an exploratory analysis of inflammatory cytokines and chemokines measured in a subset of 26 children aged 0–18 years with TBI and 21 controls. TBI severity was determined by GCS. The functional outcome was measured via the GOS-E score at 6 weeks and 3, 6, 9, and 12 months post-injury. Serum samples were analyzed for ICAM-1, VCAM-1, SAA, CRP, IFN-g, IL-10, IL-12p70, IL-13, IL-1b, IL-2, IL-4, IL-6, IL-8, TNF-a, TNF-b, eotaxin, eotaxin-3, IP-10, MCP-1, MCP-4, MDC, MIP-1a, MIP-1b and TARC. Levels of IL-6, IL-10, IL-13, IL-16, MDC, and GM-CSF were increased, and IFN-γ, IL-5, IL-8, and eotaxin-3 were decreased at enrollment when compared with controls. Elevated IL-6 and IL-10 at enrollment were associated with severe TBI (AUC of 1, *p* = 0.0002 and *p* = <0.0001, respectively). IL-6, IL-10, IL-16, and TNF-β at enrollment and IL-5 at 24 h were elevated in children with unfavorable outcomes, with an AUC > 0.8, suggesting biomarker potential. Our data indicate that several cytokines and chemokines measured after TBI may aid in the assessment of pTBI severity and prognosis. IL-6, IL-10, and IL-16 may show potential as biomarkers for pTBI severity and outcomes.

## 1. Introduction

Globally, traumatic brain injury (TBI) is one of the leading causes of death and disability for adults and children alike, with over 2000 TBI-related deaths per year occurring in children under the age of 17 in the United States alone and over 61% of patients with moderate–severe TBI experiencing disability from these injuries [1,2,3]. It results in individual, family, and societal dysfunction with potentially significant but thus far unquantifiable economic impact [4,5,6,7]. While neurological symptoms can vary, the degree of inflammation, immune dysregulation, and individual neuroinflammatory variation impact patient outcomes [8,9,10].

The search for biomarkers that can be utilized as indicators of TBI severity and prognosis with greater accuracy is ongoing [11,12,13]. In addition, the ability of these biomarkers to detect more subtle differences in the spectrum from concussion to severe TBI and to predict positive CT findings requires additional studies to verify their validity [11]. Some of the most studied biomarkers have been those of neuronal injury, such as ubiquitin c-terminal hydrolase-L1 (UCHL-1), glial fibrillary acidic protein (GFAP), S100-B, myelin basic protein (MBP), neuron-specific enolase (NSE), Tau, and *p*-Tau, which are valuable indicators of TBI severity and prognosis [14,15,16,17,18]. Others have included markers of blood-brain barrier injury, components of the hypothalamic-pituitary axis as indicators of the acute stress response, enzymes released in the setting of cellular dysfunction, and markers of local and systemic inflammation [11,19,20].

The inflammatory response elicited in TBI involves local cerebral production and activation of cytokines and chemokines; endothelial activation; microglial activation; and the migration of systemic neutrophils, lymphocytes, and monocytes into the injured brain [8,21].

Although current management aims at preventing secondary injury through supportive care, therapies to limit acute and chronic inflammatory responses are still being investigated [22,23]. This balance of proinflammatory and anti-inflammatory cytokines may help guide patients who benefit the most from interventions that target excessive inflammation, leading to poor neurologic outcomes.

This study aimed to expand the literature on serum markers of inflammation in children by analyzing a subset of pediatric patients with TBI to determine whether specific inflammatory markers could be valuable biomarkers for predicting TBI severity or outcome.

## 2. Materials and Methods

### 2.1. Study Design and Setting

This prospective observational cohort study was conducted in the pediatric intensive care units (ICUs) of two academic centers in Florida, USA, from February 2017 to February 2024. Approval was obtained from the Institutional Review Boards at the University of Florida (IRB 201600237) and the University of Miami (IRB 20210352). Informed consent was obtained from the participants or their authorized representatives within 72 h of admission. At the University of Miami, blood samples were collected only after parental consent was obtained. This study adhered to the Strengthening the Reporting of Observational Studies in Epidemiology (STROBE) reporting guidelines for cohort studies [11].

### 2.2. Participants

Children aged 0–18 years who were admitted to the pediatric hospital floors, pediatric ICU, trauma ICU, surgical ICU, or neurosurgical ICU with a diagnosis of TBI were eligible for participation. Patients with severe psychiatric disorders (per DSM-V), preexisting neurodevelopmental disorders, insufficient data for statistical analysis, or pregnancy were excluded from the study. Controls with similar demographics were recruited from the emergency department, pediatric floor, outpatient surgeries, and outpatient sedation services, excluding those with a history of TBI or acute neurological or psychiatric conditions. Severity was determined by the worst Glasgow Coma Scale (GCS) score documented in the first 6 h of admission to better assess the impact of primary injury rather than changes from secondary neurologic injury. Outcomes were evaluated based on the Glasgow Outcome Scale Extended for Pediatrics (GOS-E Peds).

TBI-Common Data Elements (TBI-CDEs), self-reported demographic information, and detailed clinical assessments were collected, including a seven-day compilation of relevant critical care information. In addition, the TBI-CDEs Biospecimens and Biomarkers Working Group Consensus guidelines for serum preparation were followed [24]. Blood samples for genetic and proteomic analysis were collected at enrollment, 24, and 48 h postinjury from pTBI patients, whereas samples from controls were collected at enrollment. Samples collected at enrollment were limited to those obtained within the first 12 h after injury.

The blood sampling volume was a total of 2 cc/kg, up to a maximum of 5 mL for children aged 0–4 years and 10 mL for children aged 5–18 years. Red top SST BD Vacutainer^®^ Plus tubes (Avantor, Radnor, PA 19087, USA) were used for blood collection. Following IRB-approved protocols, deidentified samples were transferred from the University of Florida to the University of Miami for analysis. The serum was stored at −80 °C after being centrifuged in 0.5 μL aliquots.

There were a total of 87 patients and 25 controls throughout the period of study. To determine the potential benefit of testing cytokines and chemokines in pediatric TBI, an exploratory sample of 22 pTBI patients with serum samples at enrollment, 24 and 48 h were chosen alongside 16 available controls.

### 2.3. Enhanced Chemiluminescent Immunoassays (ECLIA)

Cytokine and chemokine levels were determined via Meso Scale Discovery (MSD) Assays as described by Scott et al. [25] The Proinflammatory Panel 1 (human) Kit V-PLEX included IFN-γ, IL-1β, IL-2, IL-4, IL-6, IL-8, IL-10, IL-12p70, IL-13 and TNF-α. The Cytokine Panel 1 Human Kit V-PLEX included GM-CSF, IL-1α, IL-5, IL-7, IL-12/IL-23p40, IL-15, IL-16, IL-17A, TNF-β and VEGF-A. The V-PLEX Chemokine Panel 1 Human Gen. B Kit included eotaxin, eotaxin-3, IP-10, IL-8 (HA), MCP-1, MCP-4, MDC, MIP-1α, MIP-1β and TARC. Finally, the V-PLEX Vascular Injury Panel 2 Human Kit included CRP, ICAM-1, SAA, and VCAM-1. Of note, though a total of 34 markers were tested, only 24 markers yielded results within a detectable range for subsequent analysis. All V-PLEX Meso Scale Discovery Kits were obtained from 1601 Research Boulevard, Rockville, MD 20850-3173, USA.

### 2.4. Outcome Measures

The primary outcome was functional recovery, measured by the GOS-E score. This scale ranges from 1, denoting complete recovery, to 8, indicating death. Scores were dichotomized into favorable outcomes (GOS-E scores ≤ 4) and unfavorable outcomes (GOS-E scores ≥ 5). The outcomes were measured at 2 weeks, 6 weeks and, 3, 6, 9, and 12 months post-injury. To optimize data completeness, these data were grouped into three periods: 2–6 weeks, 3–6 months, and 9–12 months.

### 2.5. Statistical Analysis

A sample of 22 pTBI patients and 16 controls were selected based on serum and data availability. Only patients with serum samples at 2 or more time points (0, 24, and 48 h), a GCS score at admission, and more than one outcome assessment by the GOS-E Peds within 12 months were included in our analysis. Final analysis was performed with the average outcome by GOS-E Peds over the 12-month period.

A sample size of 20 per group was determined to be of sufficient power (1-β) of 0.80 based on an alpha (α) of 0.05 (two-tailed). Frequencies and percentages are reported for categorical variables. Associations between variables were assessed via the *χ*^2^ test or Fischer’s exact test for categorical variables or the Kruskal-Wallis test for continuous variables. The majority displayed a nonparametric distribution; therefore, IQR ranges were utilized with outliers excluded via robust regression and outlier removal (ROUT). The comparison of the serum cytokine and chemokine levels between the pTBI patients and the controls was performed via one-way ANOVA. Nonparametric tests were applied when the data were not normally distributed. The significance of the *p* values was set at *p* < 0.05 for all the statistical tests.

Analysis was performed both individually for Mild, Moderate and Severe TBI initially. Due to differences in sample size between analytes with outlier removal or undetectable levels for some cytokine analyses, TBI severity was grouped into Mild/Moderate and then Severe for additional grouped analyses.

For those cytokines and chemokines that reached statistical significance, performance as a diagnostic marker was assessed via the area under the receiver operating characteristic curve (AUROC) for sensitivity (SN), specificity (SP), and likelihood ratio (LR), with AUROC values categorized as poor (<0.7), fair (0.7–0.8), good (>0.8–0.9), or excellent (>0.9). The predictive values and assay accuracies were also calculated. Missing data were not imputed. The initial data processing was organized via Microsoft Excel (Version 16). All the statistical analyses were conducted via GraphPad Prism (Version 10).

## 3. Results

### 3.1. Demographics of Pediatric TBI Patients and Controls

**Figure 1:**
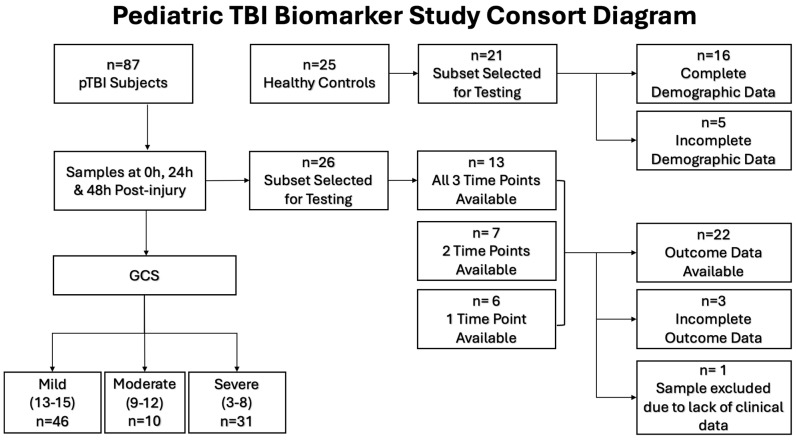
Consort diagram showing patient population selection for cytokine and chemokine analysis.

This analysis included 22 pTBI patients and 16 controls (Figure 1). There were no statistically significant differences between the two groups regarding age, sex, or ethnicity. Among the patients with TBI, 10 patients had severe TBI (48%), 5 had moderate TBI (24%), and 7 had mild TBI (33%). Notably, those with mild TBI had more severe illness based on the illness severity score (ISS). Despite the predominance of severe TBI patients, favorable outcomes based on the GOS-E score were noted in most patients (*n* = 14, 67%) (Table 1).

Controls in our study were selected both from inpatient services, outpatient procedural areas and the emergency room. It should be noted that 9 of the 16 patients (56%) of this group did have evidence of a mild infectious or inflammatory disease process (See Supplementary Materials). The levels of cytokines and chemokines analyzed maintained statistical significance regardless of comparison with those controls with underlying inflammation and those without.

### 3.2. Variation in Inflammatory Cytokines and Chemokines in Pediatric TBI Patients

**Figure 2:**
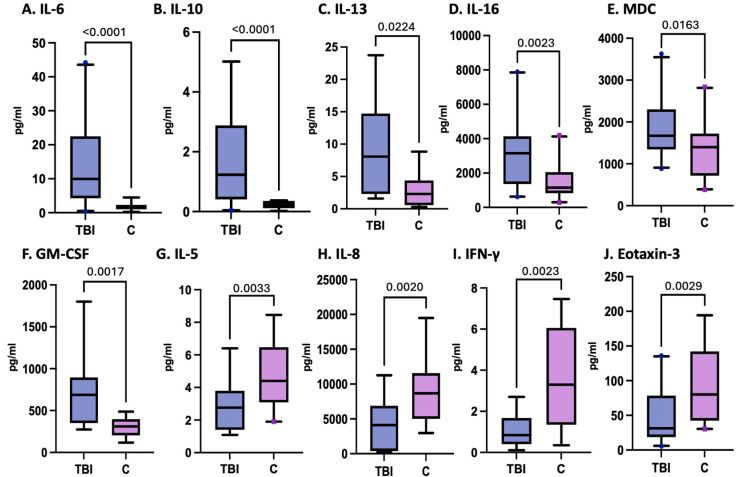
Concentrations of serum cytokines and chemokines in postinjury pTBI patients at enrollment versus controls across all injury severity levels determined by the GCS.

In the subgroup of patients assessed, the levels of IL-6, IL-10, IL-13, and IL-16 and the chemokines MDC and GM-CSF at enrollment were significantly greater in pTBI patients than in controls, as shown in Figure 2. On the other hand, the levels of IL-5, IFN-γ, IL-8, and eotaxin-3 were lower in pTBI patients than in controls at enrollment. Although MDC, IP-10, and VCAM-1 differed significantly in their levels compared to controls, the receiver operating characteristic (ROC) curves did not significantly differ.

Figure 2 Box plots depict statistically significant differences in the serum cytokine and chemokine levels in pg/mL after pTBI measured at enrollment within all TBI severity categories compared with those in the control samples. Graphs A–F represent inflammatory mediators increased in pTBI patients relative to controls. Graphs G–J represent inflammatory mediator decreases in pTBI patients relative to controls. [A] IL-6 levels at 0 h. [B] IL-10 levels at 0 h. [C] IL-13 levels at 0 h. [D] IL-16 levels at 0 h. [E] MDC levels at 0 h. [F] GM-CSF levels at 0 h. [G] IL-5 levels at 0 h. [H] IL-8 levels at 0 h. [I] IFN-γ levels at 0 h. [J] Eotaxin-3 levels at 0H. Whiskers depict the 5th and 95th percentiles. The line within the box represents the median from the dataset, while the dots denote outlier values. The numbers above the connecting lines represent statistically significant *p* values obtained via the Kruskal-Wallis test.

### 3.3. Cytokines and Chemokines as Biomarkers of Pediatric TBI

**Figure 3:**
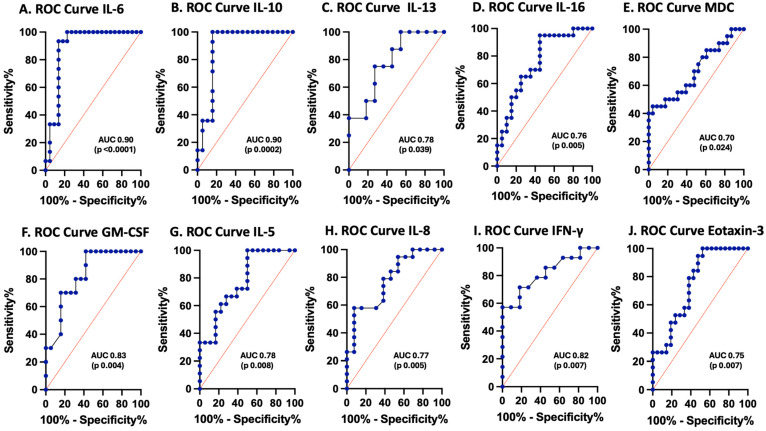
Receiver operating characteristic (ROC) curves for serum inflammasome proteins in postinjury pTBI patients versus controls across all injury severity levels determined by the GCS. Blue lines represent the data driven ROC curve. Red lines indicate performance expected by chance.

The biomarker potential of each analyte, including IL-6, IL-10, IL-13, IL-16, MDC, GM-CSF, IL-5, IFN-γ, IL-8, and eotaxin-3, measured at enrollment was assessed by calculating the ROC curves for those inflammatory cytokines and chemokines that demonstrated statistically significant differences between pTBI patients and controls (Figure 3). IL-6 (100% sensitivity, 77% specificity) and IL-10 (100% sensitivity, 84% specificity) had AUCs of 0.9 each, whereas GM-CSF (70% sensitivity, 84% specificity) and IFN-γ (71% sensitivity, 82% specificity) had AUCs of 0.8, as shown in Table 2.

Figure 3 ROC curves for serum cytokines and chemokines in postinjury pTBI patients at enrollment versus controls across all injury severity levels. Graphs A–F represent inflammatory mediator increases in patients with pTBI. Graphs G–J represent inflammatory mediator decreases in patients with pTBI. [A] ROC curve for IL-6. [B] ROC curve for IL-10. [C] ROC curve for IL-13. [D] ROC curve for IL-16. [E] ROC curve for MDC. [F] ROC curve for GM-CSF. [G] ROC curve for IL-5. [H] ROC curve for IL-8. [I] ROC curve for IFN-γ. [J] ROC curve for eotaxin-3. Sensitivity is represented on the *y*-axis, and specificity is represented on the *x*-axis.

### 3.4. Variations in Inflammatory Cytokines and Chemokines Associated with pTBI Severity Based on the GCS

**Figure 4:**
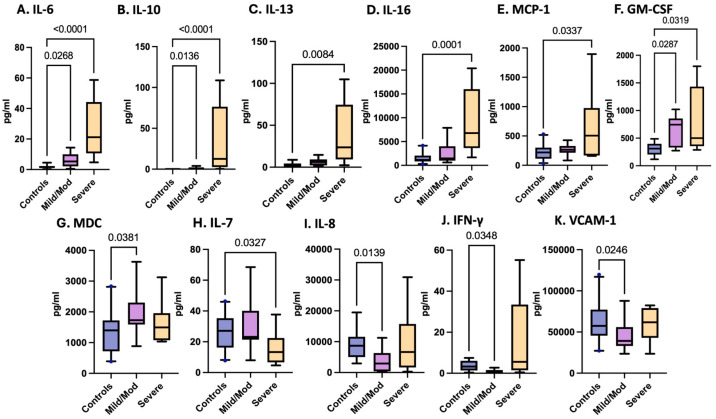
Concentrations of serum cytokines and chemokines in postinjury pTBI patients as categorized by TBI severity as determined by the Glasgow Coma Scale (GCS).

Adjusted for TBI severity at enrollment, levels of IL-6, IL-10, IL-13, IL-16, MCP-1, and MDC were significantly elevated in pTBI patients. In contrast, IL-7 was reduced in children with severe TBI at enrollment compared with controls, while IL-8, IFN-γ, and VCAM-1 were decreased in patients with mild to moderate TBI relative to controls (Table 3 and Figure 4).

Figure 4 Box plots depict statistically significant serum cytokine and chemokine levels at enrollment in pg/mL in those with combined mild/moderate TBI and severe TBI, as determined by the GCS. Graphs A–G represent inflammatory mediators increased in pTBI patients relative to controls. Graphs H–K represent inflammatory mediator decreases in pTBI patients relative to controls. [A] IL-6 [B] IL-10 [C] IL-13 [D] IL-16 [E] MCP-1 [F] GM-CSF [G] MDC [H] IL-7 [I] IL-8 [J] IFN-γ [K] VCAM-1. Whiskers depict the 5th and 95th percentiles. The numbers above the connecting lines represent statistically significant *p* values obtained by the Kruskal-Wallis test.

### 3.5. Cytokines and Chemokines as Biomarkers of Pediatric TBI Severity

**Figure 5:**
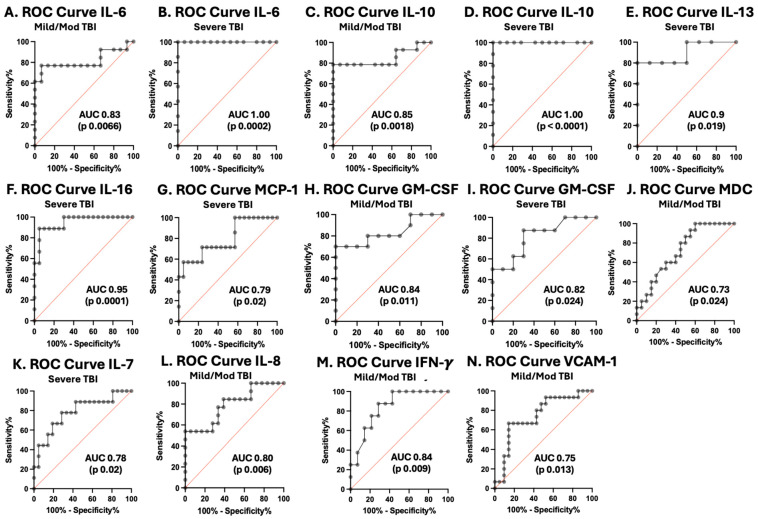
Receiver operating characteristic curves for serum cytokines and chemokines in postinjury pTBI patients versus controls across all injury severity levels determined by the GCS. Grey lines represent the data driven ROC curve. Red lines indicate performance expected by chance.

ROC curves were plotted for each cytokine and chemokine that significantly varied with TBI severity, including IL-6, IL-10, IL-13, IL-16, MCP-1, GM-CSF, MDC, IL-7, IL-8, IFN-γ, and VCAM-1 (Figure 5 and Table 4). IL-6 and IL-10 had the best performance as biomarkers of severe TBI, with an AUC of 1.0 (sensitivity 100%, specificity 100%). IL-13 had an AUC of 0.9 (sensitivity of 80%, specificity of 100%), and IL-16 had an AUC of 0.95 (sensitivity of 100%, specificity of 70%) as a predictor of severe pTBI.

Figure 5 ROC curves for serum cytokines and chemokines in postinjury pTBI patients versus controls across all injury severity levels determined by the GCS. Graphs A–J represent inflammatory mediators increased in pTBI patients at enrollment compared with controls separated by GCS category. Graphs K–N represent inflammatory mediator decreased in pTBI patients relative to controls separated by GCS category. [A] ROC curve for IL-6 in mild/moderate pTBI. [B] ROC curve for IL-6 in severe pTBI. [C] ROC curve for IL-10 in mild/moderate pTBI. [D] ROC curve for IL-6 in severe pTBI. [E] ROC curve for IL-13 severe pTBI. [F] ROC curve for IL-16 in severe pTBI. [G] ROC curve for MCP-1 in severe. [H] ROC curve for GM-CSF in mild/moderate pTBI. [I] ROC curve for GM-CSF severe pTBI. [J] ROC curve for MDC in mild/moderate pTBI. [K] ROC curve for IL-7 in severe pTBI. [L] ROC curve for IL-8 in mild/moderate pTBI. [M] ROC curve for IFN-γ mild/moderate pTBI. [N] ROC curve for VCAM-1 in mild/moderate pTBI. Sensitivity is represented on the *y*-axis, and specificity is represented on the *x*-axis.

**Figure 6:**
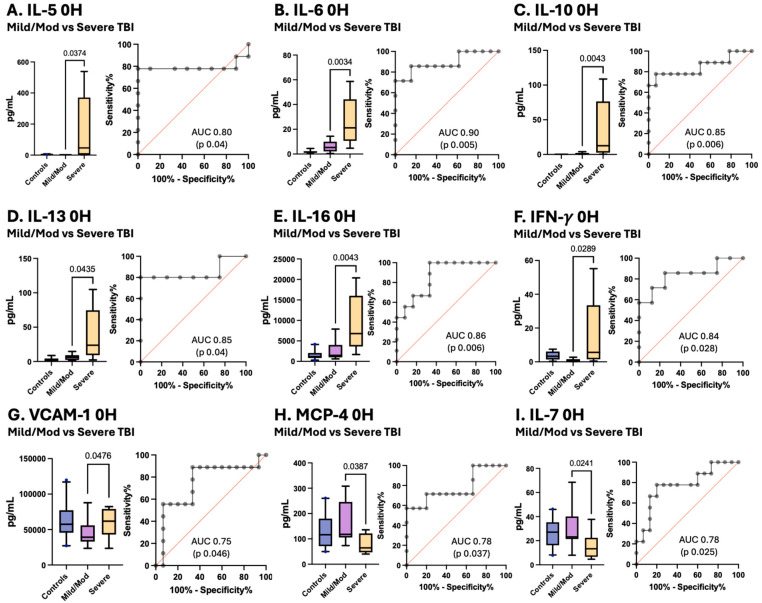
Concentrations of serum cytokines and chemokines in pTBI patients with mild/moderate pTBI versus severe TBI based on the GCS score along with corresponding receiver operating characteristic (ROC) curves. Grey lines represent the data driven ROC curve. Red lines indicate performance expected by chance.

Notably, for IL-5, IL-6, IL-10, IL-13, Il-16, IFN-γ, and VCAM-1, the serum levels at enrollment increased with increasing TBI severity, and the MCP-4 and IL-7 levels decreased with increasing TBI severity, facilitating differentiation between mild/moderate pTBI patients and severe pTBI patients (Figure 6).

Figure 6 Box plots depict statistically significant differences in the serum cytokine and chemokine levels in pg/mL between pTBI patients with mild/moderate TBI and those with severe TBI measured at enrollment. Graphs A–G show that the levels of inflammatory mediators increased in patients with increasing severity of TBI. Graphs H–I represent inflammatory mediators decreased in patients with decreasing TBI severity. [A] IL-5 [B] IL-6 [C] IL-10 [D] IL-13 [E] IL-16 [F] IFN-γ [G] VCAM-1 [H] MCP-4 [I] and IL-7. Whiskers depict the 5th and 95th percentiles. The line within the box represents the median from the dataset. The numbers above the connecting lines represent statistically significant *p* values obtained via the Mann-Whitney test. For the ROC curves, the sensitivity is represented on the *y*-axis, and the specificity is represented on the *x*-axis.

### 3.6. Cytokines and Chemokines as Biomarkers of Pediatric TBI Outcomes

**Figure 7:**
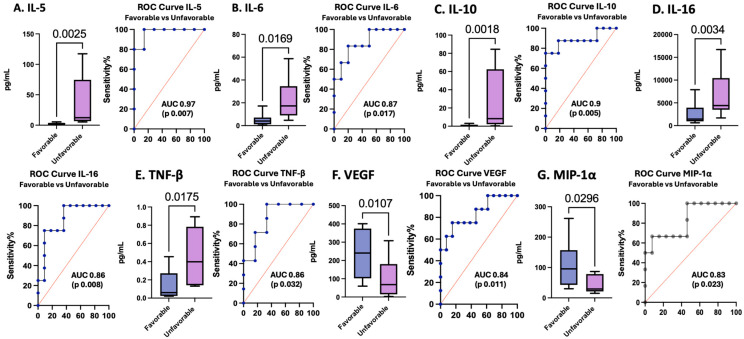
Concentrations of serum cytokines and chemokines in pTBI patients with favorable versus unfavorable outcomes across all injury severities based on the GOS-E score along with corresponding receiver operating characteristic curves. Blue lines represent the data driven ROC curve. Red lines indicate performance expected by chance.

The GOS-E score is a frequently utilized measure of outcomes after TBI. In this cohort, IL-5 levels measured at 24 h and IL-6, IL-10, IL-16, and TNF-β levels measured at enrollment were elevated in pTBI patients who had unfavorable outcomes, whereas VEGF and MIP-1α levels were decreased in those with unfavorable outcomes. IL-5 measured at 24 h performed the best as a biomarker for unfavorable outcomes, with an AUC of 0.97 (sensitivity 100%, specificity 86%). All other proteins performed well as biomarkers of outcomes: IL-6, IL-10, IL-16, TNF-β, VEGF, and MIP-1α (Table 5 and Figure 7).

Figure 7 Box plots depict statistically significant serum cytokine and chemokine levels in pg/mL in those with favorable versus unfavorable outcomes. Graphs A–E represent inflammatory mediator increases in patients with unfavorable outcomes. Graphs F–G represent inflammatory mediator decreases in patients with unfavorable outcomes. [A] IL-5 levels in pTBI patients with favorable versus unfavorable outcomes at 24 h. [B] IL-6 levels in pTBI patients with favorable versus unfavorable outcomes at 0 h. [C] IL-10 in pTBI patients with favorable versus unfavorable outcomes at 0 h. [D] IL-16 in pTBI patients with favorable versus unfavorable outcomes at 0 h. [E] TNF-β in pTBI patients with favorable versus unfavorable outcomes at 0 h. [F] VEGF in pTBI patients with favorable versus unfavorable outcomes at 0 h. [G] MIP-1α in pTBI patients with favorable versus unfavorable outcomes at 0 h. Whiskers depict the 5th and 95th percentiles. The line within the box represents the median from the dataset. The numbers above the connecting lines represent statistically significant *p* values obtained via the Kruskal-Wallis test. For the ROC curves, the sensitivity is represented on the *y*-axis, and the specificity is represented on the *x*-axis.

### 3.7. Cytokines and Chemokines as Potential Predictors of Positive Computed Tomography Findings

**Figure 8:**
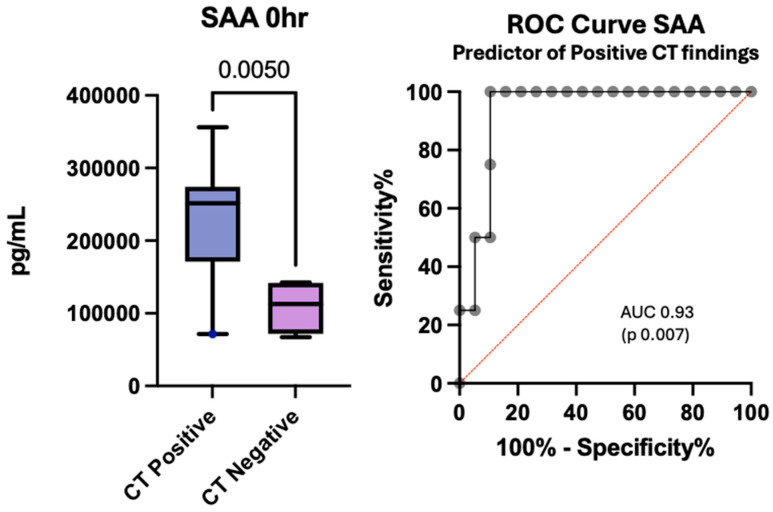
Concentration of serum SAA at enrollment in patients with positive CT findings versus negative CT findings after pediatric traumatic brain injury and the associated receiver operating curve.

The ability to screen patients with TBI who need neuroimaging to both limit unnecessary radiation exposure and facilitate judicious utilization of resources is an important quest. In our cohort of patients, serum amyloid A (SAA) was significantly greater in those with positive CT findings than in those with negative CT findings (*p* 0.005) and was an excellent biomarker in this setting (AUC 0.93, *p* 0.007) (Figure 8). Despite the association between SAA and CT findings, there was no statistically significant difference in SAA levels based on injury severity (*p* 0.35) or when compared in those with favorable and unfavorable outcomes (*p* 0.78). Patients with higher interferon-gamma (IFN-γ) and IL12/IL-23p40 levels were more likely to have negative CT findings (*p* values of 0.03 and 0.02, respectively); however, the ROC curves did not significantly differ.

The box plot depicts statistically significant SAA levels in pg/mL. Whiskers depict the 5th and 95th percentiles. The line within the box represents the median from the dataset. The number above the connecting line represents a statistically significant *p*-value obtained by the Kruskal-Wallis test. The associated ROC curve shows the sensitivity on the *y*-axis with the specificity on the *x*-axis.

## 4. Discussion

Many individual cytokines and chemokines have been studied in murine models and subsequently evaluated in the clinical setting [26,27,28]. Multiplex analyses for profiling cytokines and chemokines have been increasingly utilized to understand the inflammatory process after TBI and to determine their biomarker potential. Although much of this work has been done in the adult population [29], pediatric data continue to emerge [30]. This is particularly important due to the known variation in neurologic, immunologic, and even microbiologic maturity in children compared with adults and the variation that occurs at each stage of childhood development and across the aging adult population [30,31,32].

Buttram et al. utilized multiplex analysis of the CSF of 36 children with severe TBI and reported that the levels of IL-1β, IL-6, IL-12p70, and IL-10 and the chemokines IL-8 and MIP-1α were increased after TBI compared with those in controls [33]. Berger et al. performed multiplex analysis to measure 44 cytokines and chemokines in the serum of 16 infants with mild inflicted TBI (ITBI) compared with a group of controls [34]. Although most of these markers were within undetectable limits, there was a significant difference in levels between patients with ITBI when compared with controls. Specifically, metallopeptidase-9 (MMP 9), hepatocyte growth factor (HGF), fibrinogen, and IL-6 were increased in patients with ITBI while intracellular adhesion molecule (ICAM), vascular cellular adhesion molecule (VCAM), IL-12, eotaxin, and tumor necrosis factor-2 (TNFR2) were decreased in those with ITBI [34].

Our preliminary study of cytokines and chemokines in this cohort of pediatric patients helps expand the growing body of literature on inflammatory patterns after TBI. Our findings reveal significant variation in several cytokines and chemokines involved in the response within the first 24 h after injury, underscoring their potential value in assessing pTBI severity and outcome.

Our data demonstrated that the levels of IL-6, IL-10, IL-13, IL-16, MDC, and GM-CSF were greater within the first 24 h of injury in pTBI in keeping with a robust early inflammatory response. This finding aligns with previous data showing the elevation of all these cytokines after TBI as anticipated components of the inflammatory response [17,29,33,34,35,36]. Notably, although elevated after TBI in some adult studies, IL-13, IL-16, MDC, and GM-CSF have not been detected or often fail to reach statistical significance in pediatric data [34,35,36]. However, the ECLIA technology used in the present study provides the sensitivity necessary to reliably measure these cytokines. The elevation in IL-6 is particularly unsurprising, as it is one of the first proinflammatory mediators to increase after injury and aids in stimulating the subsequent inflammatory cascade [37,38]. Pediatric studies have consistently shown elevated IL-6 after TBI, regardless of severity [34,39,40,41]. An increase in IL-10 is also expected, as it is one of the main mediators responsible for resolving the inflammatory response [37,42].

Conversely, the levels of IFN-γ, IL-5, IL-8, and eotaxin-3 were lower at enrollment in pTBI patients than in controls. While data concerning these specific cytokines in the setting of TBI are limited, variability has been noted to be dependent on the severity and timing of injury. For example, increases in IFN-γ have been observed after mild TBI, but decreased levels are observed in severe TBI patients compared with controls [39]. The presence of more severely ill mild pTBI patients in our cohort may explain the finding of lower IFN-γ levels in pTBI patients than in controls. Decreased levels of eotaxin-3 within the first 24 h after pTBI are also in keeping with the current literature but have demonstrated sustained elevation thereafter, suggesting significant time-based variation in serum levels that requires further investigation in the pediatric population [43]. The decreases in the levels of IL-5 and IL-8 contradict the published data, which noted increases in these markers within the first 24 h after injury [32,36,44]. This variation in IL-8 after TBI may be related to time-based fluctuations in IL-8 levels after injury, where an early initial increase has been shown to occur [45]. Ryan et al. reported similar findings in pediatric patients with mild TBI, where a decrease in IL-8 compared with that in controls was noted and sustained for up to 2 weeks postinjury [39]. Future studies at various time intervals are likely needed to fully delineate the pattern of change in IL-8 after injury.

We also observed that IL-6, IL-8, IL-10, IL-13, IL-16, MCP-1, GM-CSF, and MDC distinguish patients with pTBI across injury severities as determined by the GCS. IL-5, IL-6, IL-10, IL-13, IL-16, IFN-γ and VCAM-1 levels increase with increasing TBI severity, with IL-6, IL-10, IL-13, and IL-16 being excellent biomarkers of severe pTBI. Elevated IL-5, IL-6, IL-10, IL-16, and TNF-β levels increase the likelihood of unfavorable outcomes and perform well as biomarkers in this setting. The finding of IL-5 elevation in those with more severe injury and its association with unfavorable outcomes differs from the findings of the TRACK TBI study, which revealed statistically significant elevation in those with TBI but did not find an association with outcome or severity [36]. Elevated IL-13, a predictor of greater injury severity, has previously been noted in murine models and adult data, with the potential to distinguish between mild-to-severe TBI but has failed to show significance in prior pediatric data [29,46,47]. These cytokines may benefit from further study.

According to previous studies, the levels of IL-6 and IL-10 are elevated after TBI and are independently associated with increased severity and unfavorable outcomes in adult and pediatric data [20,29,35,36,48,49]. In addition, when utilized alongside UCH-L1, they improve the accuracy of severity and outcome prediction [48]. An increase in IL-8 in patients with greater injury severity has been previously noted [39]. Although we did not observe a correlation with unfavorable outcomes in this pediatric cohort, IL-8 was found to predict unfavorable outcomes in both adult and pediatric data. Persistent elevation in IL-8 has been noted to indicate blood–brain barrier dysfunction and to predict persistent fatigue, unfavorable outcomes and even mortality after TBI [41,50,51,52]. When combined with the GCS for prognostication, IL-8 improves the sensitivity and specificity to 100% and 96%, respectively [39,53].

VCAM-1, which has been found to be useful as a biomarker of pTBI, was decreased in patients with mild–moderate pTBI compared with controls but increased in those with severe pTBI, reflecting time- and injury-specific variations that require further elucidation [34]. IL-7 was decreased in patients with severe pTBI, but although it was decreased in adult TBI patients, it has not previously been associated with injury severity or outcome and has not been shown to be statistically significant in pediatric patients [33,34,36].

In our evaluation of biomarkers for the prediction of positive CT findings, SAA was the only marker that showed both statistical significance and good biomarker function, which corroborates data from the TRACK-TBI study [36]. Though the reason for its lack of correlation to TBI severity and Outcomes is unclear, our sample size may not be sufficient to delineate the full extents of its association. The determination of children who benefit the most from CT imaging after head injury continues to be a challenge [54,55]. Although SAA is a nonspecific marker of inflammation, it may be worth exploring its utility alongside current clinical criteria to determine those who will most benefit from neuroimaging. It is notable that the International Mission for Prognosis and Analysis of Clinical Trials in TBI (IMPACT) prognostic calculator, which incorporates clinical data on the injury, found improved predictive value when combined with Glial Fibrillary Acidic Protein (GFAP) levels. The possibility of combining SAA with GFAP or other brain-specific biomarkers to enhance risk stratification and identify children who may safely forgo imaging is an important area for additional investigation.

Cytokine evaluation is also important in identifying targets for therapeutic interventions. Prior attempts to limit the inflammatory response in the setting of TBI revealed that widespread inhibition of inflammation with corticosteroids led to greater harm than benefit [23,56,57]. Pinpointing specific time points in the inflammatory process to limit but not eliminate the chronic inflammatory process may produce better results [23].

The inflammatory markers most consistent with the presence of TBI, increased disease severity, and prediction of unfavorable outcomes in our cohort were IL-6, IL-10, and IL-16. IL-16 has not previously been found to be significant after pediatric TBI, making this a novel finding [33,34]. IL-6 and IL-10 were noted to be excellent discriminators of severe pTBI, with an AUC of 1.0 (*p* < 0.0001). While these cytokines tend to be nonspecific markers of the inflammatory response, they may be useful along with more specific markers of neuronal injury, as observed prior to their use alongside the GCS or UCHL-1, and may aid in classifying TBI severity, prognosis, and risk of in-hospital mortality [48,53].

Currently, monoclonal antibodies have been used to inhibit the action of IL-6 to limit an excessive inflammatory response and its potentially detrimental effects [58]. These include both direct IL-6 inhibitors and IL-6 receptor inhibitors. Although mostly used in the setting of rheumatologic conditions, its use for hyperinflammatory states induced by various disease processes, such as COVID-19, has increased, with promising results, so its use is now considered the standard of care [59,60]. Mouse models have been utilized to determine the impact of various inhibitors of IL-6 and IL-10 after TBI; however, data concerning their benefits are conflicting [61,62]. Thus, IL-6 continues to be a potential area of study for improving outcomes after TBI in the future.

## 5. Limitations

While this study was beneficial in its ability to corroborate the current literature revealing increases in proinflammatory and anti-inflammatory cytokines and chemokines after pediatric TBI, this small sample size, made even smaller by incomplete data, was utilized for exploratory evaluation. It is therefore insufficient to draw generalizable conclusions. Samples at the 24- and 48-h time points were also limited; therefore, the variation in inflammatory markers across time points could not be completely elucidated.

We also note that our sample had a greater proportion of patients with mild TBI who had higher illness severity scores, which may have led to higher cytokine levels and therefore a lack of statistical significance between TBI severity groups than may be ordinarily seen. In our cohort, we examined whether the presence of polytrauma correlated with cytokine levels. There was no significant association between polytrauma status and the concentrations of the measured inflammatory markers. Patients with isolated TBI and those with TBI plus polytrauma demonstrated comparable cytokine profiles across all analytes tested.

Importantly, the distribution of polytrauma within our cohort was limited, and the study was not powered specifically to detect differences driven by peripheral trauma. Therefore, while no correlations were observed, we acknowledge that larger, more targeted studies may be required to fully delineate the impact of extracranial injury on systemic inflammation in pediatric TBI.

## 6. Conclusions

Our study demonstrated possible correlations between elevated levels of IL-6 and IL-10 and the presence of pTBI, as well as a possible correlation between IL-5, IL-7, IL-13 and IL-16 with injury severity and outcome, which has not been previously reported in pediatric data. Other biomarkers have shown value in the discrimination of severity and outcome after pediatric TBI, but understanding the variation between adult and pediatric inflammatory responses will require standardization of testing methodology, reference values, and side-by-side comparisons so that temporal trends in management do not create bias in comparisons between groups. A better understanding of the inflammatory processes after pTBI could help researchers identify key cytokines and chemokines that could be targets for therapy in clinical studies [24]. We plan to utilize this information to expand our current analysis with greater detail on cytokine analysis. An expansion of this analysis to also include comparison with other brain injury biomarkers will also be important in combining biomarkers that have already proven useful when combined with clinical data in helping to determine the need for imaging and enhanced identification of pTBI severity and prognostication.

## Figures and Tables

**Table 1:** Demographic Characteristics of Pediatric Patients with Traumatic Brain Injury compared with controls in the evaluation of Serum Cytokines & Chemokines.

Characteristic	Controls		Pediatric Traumatic Brain Injury Patients			
			Favorable Outcome		Unfavorable Outcome	
	(*n* = 16)		(GOS-E Peds ≤ 4) (*n* = 14)		(GOS-E Peds ≥ 5) (*n* = 8)	
Age in years, mean (SD)	8.2	5.5	4.0	6.1	2.6	3.6
Gender, *n* (%)						
Female	7	44%	4	29%	6	50%
Male	8	50%	10	71%	2	17%
Race, *n* (%)						
White	7	50%	7	50%	3	38%
African American	0	0%	4	29%	3	38%
Unknown	4	29%	3	21%	2	25%
Payer status, *n* (%)						
Medicaid	4	25%	7	44%	5	63%
Other	8	50%	5	31%	3	38%
BMI, mean (SD)			19.2	5.7	21.0	8.1
GCS, *n* (%)						
Severe (GCS:3–8)	NA		4	29%	6	75%
Moderate (GCS:9–12)			3	21%	2	25%
Mild (GCS:13–15)			7	50%	0	0%
ISS Score, *n* (%)						
Minor (1–8)	NA		0	0%	0	0%
Moderate (9–15)			0	0%	0	0%
Serious (16–24)			3	21%	0	0%
Severe (25–49)			9	64%	4	50%
Critical (50–75)			1	7%	4	50%
PRISM Score, *n* (%)						
5–9	NA		4	18%	1	13%
10–14			3	14%	1	13%
15–19			3	14%	1	13%
20–24			1	5%	1	13%
25–29			0	0%	1	13%
30–34			0	0%	0	0%
≥35			0	0%	1	13%
Neuroimaging						
CT Positive	NA		12	86%	6	75%
CT Negative			2	14%	1	13%
Marshall Score						
1	NA		2	14%	1	13%
2			9	64%	5	63%
3			2	14%	1	13%
4			1	7%	1	13%

BMI: Body Mass Index, CT: Computed Tomography, GCS: Glasgow Coma Scale, GOS-E Peds: Glasgow Outcome Scale–Extended Pediatrics Score, ISS: Injury Severity Score, NA: Not Applicable, PRISM: Pediatric Risk of Mortality Score.

**Table 2:** Receiver operating characteristic (ROC)-derived characteristics of cytokines and chemokines in controls compared with a subset of pediatric patients with traumatic brain injury.

Biomarkers	AUROC	SE	95% CI	*p*-Value			
IL-5	0.7794	0.083	0.6167 to 0.9420	0.0081	Cutoff	(pg/mL)	>3.016
					Sensitivity	(%)	79
					Specificity	(%)	62
IL-6	0.8909	0.05837	0.7765 to 1.000	<0.0001	Cutoff	(pg/mL)	<4.576
					Sensitivity	(%)	100
					Specificity	(%)	77
IL-10	0.8872	0.06352	0.7627 to 1.000	0.0002	Cutoff	(pg/mL)	<0.3728
					Sensitivity	(%)	100
					Specificity	(%)	84
IL-13	0.7841	0.1055	0.5773 to 0.9909	0.039	Cutoff	(pg/mL)	<3.597
					Sensitivity	(%)	75
					Specificity	(%)	73
IL-16	0.7575	0.07669	0.6072 to 0.9078	0.0053	Cutoff	(pg/mL)	<1552
					Sensitivity	(%)	70
					Specificity	(%)	65
CP-IL8	0.7747	0.07739	0.6230 to 0.9264	0.0049	Cutoff	(pg/mL)	>2939
					Sensitivity	(%)	100
					Specificity	(%)	50
IFN-γ	0.8182	0.08427	0.6530 to 0.9833	0.0073	Cutoff	(pg/mL)	>1.725
					Sensitivity	(%)	71
					Specificity	(%)	82
GM-CSF	0.8263	0.07616	0.6771 to 0.9756	0.0044	Cutoff	(pg/mL)	<343.7
					Sensitivity	(%)	70
					Specificity	(%)	84
MDC	0.7011	0.08156	0.5412 to 0.8609	0.0243	Cutoff	(pg/mL)	<1307
					Sensitivity	(%)	80
					Specificity	(%)	43
Eotaxin-3	0.7519	0.07697	0.6010 to 0.9027	0.0065	Cutoff	(pg/mL)	>41.43
					Sensitivity	(%)	79
					Specificity	(%)	62

CP: Cytokine Panel, GM-CSF: Granulocyte-Macrophage Colony-Stimulating Factor, IL: Interleukin, IFN-γ: Interferon Gamma, MDC: Macrophage-derived Chemokine.

**Table 3:** Cytokine & Chemokine values in Controls vs. Patients with Traumatic Brain Injury based on Severity as Determined by Glasgow Coma Scale (GCS).

	Controls (*n* = 21)			Traumatic Brain Injury (*n* = 22)						
Biomarkers	Median (pg/mL)	S.D. (pg/mL)	IQR (pg/mL)		*n*	Median (pg/mL)	S.D. (pg/mL)	IQR (pg/mL)	Mean Rank Difference	*p*-Value
IL-6	0.2275	1.095	1.003–2.301	Severe	7	21.15	19.12	10.64–44.21	−29.91	<0.0001
IL-10	0.2234	0.1263	0.1113–0.3559	Mild/Moderate	14	1.022	1.175	0.348–1.871	−15.93	0.0183
				Severe	9	12.58	41.5	2.54–76.45	−30.99	<0.0001
IL-13	2.294	2.821	0.5675–4.376	Severe	5	23.73	40.14	9.363–74.53	−15.05	0.0076
IL-16	1150	953.4	832–2053	Severe	9	6772	6841	3632–16,008	−25.12	0.0002
CP-IL8	8668	4359	5014–11,581	Mild/Moderate	13	2917	3924	371–6314	14.47	0.0255
VCAM-1	57,406	23,059	45,579–77,190	Mild/Moderate	15	39,218	16,407	33,099–55,924	14.8	0.0486

CP: Cytokine Panel, IL: Interleukin, GM-CSF: Granulocyte-Macrophage Colony-Stimulating Factor, VCAM: Vascular Cell Adhesion Molecule.

**Table 4:** Receiver Operating Characteristic (ROC)-derived cutoff for Cytokines & Chemokines in Controls vs. a subset of pediatric patients with Traumatic Brain Injury based on Severity as Determined by Glasgow Coma Scale (GCS).

Biomarkers	TBI Severity	AUROC	SE	95% CI	*p*-Value			
IL-6	Mild/Moderate	0.8267	0.1046	0.6216 to 1.000	0.0066	Cutoff	(pg/mL)	>3.050
						Sensitivity	(%)	77
						Specificity	(%)	93
	Severe	1	0	1.000 to 1.000	0.0002	Cutoff	(pg/mL)	>4.576
						Sensitivity	(%)	100
						Specificity	(%)	100
IL-10	Mild/Moderate	0.8469	0.08355	0.6832 to 1.000	0.0018	Cutoff	(pg/mL)	>0.3905
						Sensitivity	(%)	79
						Specificity	(%)	100
	Severe	1	0	1.000 to 1.000	<0.0001	Cutoff	(pg/mL)	>0.3728
						Sensitivity	(%)	100
						Specificity	(%)	100
IL-13	Severe	0.9	0.1012	0.7016 to 1.000	0.0192	Cutoff	(pg/mL)	>12.64
						Sensitivity	(%)	80
						Specificity	(%)	100
IL-16	Severe	0.95	0.04011	0.8714 to 1.000	0.0001	Cutoff	(pg/mL)	>1603
						Sensitivity	(%)	100
						Specificity	(%)	70
CP IL-8	Mild/Moderate	0.7949	0.08314	0.6319 to 0.9578	0.0057	Cutoff	(pg/mL)	<6842
						Sensitivity	(%)	85
						Specificity	(%)	61
IL-7	Severe	0.7831	0.09633	0.5943 to 0.9719	0.0155	Cutoff	(pg/mL)	<26.24
						Sensitivity	(%)	89
						Specificity	(%)	57
VCAM-1	Mild/Moderate	0.746	0.08508	0.5793 to 0.9128	0.0129	Cutoff	(pg/mL)	<55,944
						Sensitivity	(%)	80
						Specificity	(%)	57
GM-CSF	Mild/Moderate	0.8350	0.09606	0.6467 to 1.000	0.0113	Cutoff	(pg/mL)	>343.7
						Sensitivity	(%)	80
						Specificity	(%)	70
	Severe	0.8188	0.1019	0.6189 to 1.000	0.0235	Cutoff	(pg/mL)	>346.2
						Sensitivity	(%)	88
						Specificity	(%)	70

CP: Cytokine Panel, IL: Interleukin, GM-CSF: Granulocyte-Macrophage Colony-Stimulating Factor, VCAM: Vascular Cell Adhesion Molecule.

**Table 5:** Receiver Operating Characteristic (ROC)-derived cutoff for Cytokines & Chemokines in Patients with Traumatic Brain Injury based on Favorable vs. Unfavorable Outcomes.

Biomarkers	Sample Time	AUROC	SE	95% CI	*p*-Value			
IL-5	24 h	0.9714	0.04373	0.8857 to 1.000	0.0074	Cutoff	(pg/mL)	>4.326
						Sensitivity	(%)	100
						Specificity	(%)	86
IL-6	0 h	0.8667	0.09477	0.6809 to 1.000	0.017	Cutoff	(pg/mL)	>8.135
						Sensitivity	(%)	83
						Specificity	(%)	80
IL-10	0 h	0.8864	0.09063	0.7087 to 1.000	0.005	Cutoff	(pg/mL)	>2.834
						Sensitivity	(%)	75
						Specificity	(%)	91
IL-16	0 h	0.8636	0.086	0.6951 to 1.000	0.0082	Cutoff	(pg/mL)	>3998
						Sensitivity	(%)	75
						Specificity	(%)	91
TNF-β	0 h	0.8571	0.1096	0.6423 to 1.000	0.0321	Cutoff	(pg/mL)	>0.2665
						Sensitivity	(%)	71
						Specificity	(%)	83
MIP-1α	0 h	0.8333	0.1038	0.6299 to 1.000	0.0226	Cutoff	(pg/mL)	<91.21
						Sensitivity	(%)	100
						Specificity	(%)	54
VEGF	0 h	0.8365	0.0942	0.6519 to 1.000	0.0113	Cutoff	(pg/mL)	<98.62
						Sensitivity	(%)	75
						Specificity	(%)	85

IL: Interleukin, TNF-β: Tumor Necrosis Factor Beta, MIP-1α: Macrophage Inflammatory Protein-1 Alpha, VEGF: Vascular Endothelial Growth Factor.

## Data Availability

The datasets generated and/or analyzed during the current study are not publicly available for continued collection and processing within the Pediatric TBI Consortium but are available from the corresponding authors upon reasonable request.

## Supplementary Materials

The following supporting information can be downloaded at: https://www.mdpi.com/article/10.3390/cells15010019/s1: Table S1: Diagnoses and Corresponding ICD-10 Codes of the Selected Controls; Table S2: Sensitivity, Specificity, Positive and Negative predictive values for each Cytokine & Chemokine in predicting TBI in a subset of patients with Pediatric Traumatic Brain Injury; Table S3: Sensitivity, Specificity, Positive and Negative predictive values for each Cytokine & Chemokine in predicting Mild-Moderate or Severe TBI based on Glasgow Coma Scale (GCS) in a subset of patients with Pediatric Traumatic Brain Injury; Table S4: Sensitivity, Specificity, Positive and Negative predictive values for each Cytokine & Chemokine in predicting Unfavorable Outcomes in a subset of patients with Pediatric Traumatic Brain Injury.
