# Supplementary material for: Potential Role of Serum Cytokines and Chemokines as Biomarkers of Injury Severity and Functional Outcomes Following Pediatric Traumatic Brain Injury
Source: Cells. 2025 Dec 22;15(1):19. doi: 10.3390/cells15010019 (PMC12784923; doi:10.3390/cells15010019)
Supplement: Supplementary file 1 [file cells-15-00019-s001.zip › cells-3993832-supplementary.pdf]

SUPPLEMENTARY MATERIALS

Table S1. Diagnoses and Corresponding ICD-10 Codes of the Selected Controls

Diagnosis	ICD-10 Code	ICD-10 Description
**Infectious / Inflammatory Conditions**		
Otitis Media	H66.90	Otitis media, unspecified, unspecified ear
Acute Appendicitis	K35.80	Unspecified acute appendicitis
Acute Appendicitis	K35.80	Unspecified acute appendicitis
Crohn's ileitis, flare	K50.018	Crohn’s disease of small intestine with other complication
Viral syndrome	B34.9	Viral infection, unspecified
Cellulitis of hand, right	L03.113	Cellulitis of right upper limb
Abdominal Pain, vomiting, diarrhea	R10.9, R11.10, R19.7	Unspecified abdominal pain; vomiting, unspecified; diarrhea, unspecified
Transient synovitis of the right hip	M67.351	Transient synovitis, right hip
Cellulitis of toe of left foot	L03.032	Cellulitis of left toe
**Non-Infectious / Non-Inflammatory Conditions**		
Traumatic amputation of digit of one hand without complication	S68.119A	Partial traumatic amputation of unspecified finger, initial encounter
Finger laceration, initial encounter	S61.219A	Laceration without foreign body of unspecified finger without damage to nail
Contusion of soft tissue	T14.8XXA	Other injury of unspecified body region, initial encounter
Enuresis	R32	Unspecified urinary incontinence
Flexor tenosynovitis of finger	M65.849	Other synovitis and tenosynovitis, unspecified hand
Pulmonary hypertension	I27.20	Pulmonary hypertension, unspecified

Table S2. Sensitivity, Specificity, Positive and Negative predictive values for each Cytokine & Chemokine in predicting TBI in a subset of patients with Pediatric Traumatic Brain Injury

	TBI (n)	Controls (n)	**Cutoff (pg/ml)**	**Sensitivity (%)**	**95% CI**	**Specificity (%)**	**95% CI**	**Likelihood ratio**	**Prevalence (%)**	**PPV (%)**	**NPV (%)**	**Accuracy (%)**
IL-5	13	19	> 3.016	78.95	56.67% to 91.49%	61.54	35.52% to 82.29%	2.053	41%	87%	100%	91%
IL-16	20	20	< 1552	70	48.10% to 85.45%	65	43.29% to 81.88%	2	50%	67%	68%	68%
IL-6	22	15	< 4.576	100	79.61% to 100.0%	77.27	56.56% to 89.88%	4.4	59%	87%	100%	91%
IL-13	11	8	< 3.597	75	40.93% to 95.56%	72.73	43.44% to 90.25%	2.75	58%	79%	68%	74%
IFN- γ	11	14	> 1.725	71.43	45.35% to 88.28%	81.82	52.30% to 96.77%	3.929	44%	62%	79%	69%
GM-CSF	19	10	< 343.7	70	39.68% to 89.22%	84.21	62.43% to 94.48%	4.433	66%	89%	60%	75%
IL-10	19	14	< 0.3728	100	78.47% to 100.0%	84.21	62.43% to 94.48%	6.333	58%	90%	100%	93%
MDC	23	20	< 1307	50	29.93% to 70.07%	82.61	62.86% to 93.02%	2.875	53%	77%	59%	65%
MDC	23	20	< 1815	80	58.40% to 91.93%	43.48	25.63% to 63.19%	1.415	53%	62%	66%	63%
CP-IL8	18	18	> 2939	100	82.41% to 100.0%	50	29.03% to 70.97%	2	50%	67%	100%	75%
EOTAXIN-3	21	19	> 41.43	78.95	56.67% to 91.49%	61.9	40.88% to 79.25%	2.072	53%	70%	73%	71%

CP: Cytokine Panel, GM-CSF: Granulocyte-Macrophage Colony-Stimulating Factor, IL: Interleukin, IFN-γ: Interferon Gamma, MDC: Macrophage Derived Chemokine

Table S3. Sensitivity, Specificity, Positive and Negative predictive values for each Cytokine & Chemokine in predicting Mild-Moderate or Severe TBI based on Glasgow Coma Scale (GCS) in a subset of patients with Pediatric Traumatic Brain Injury

GCS	Controls (n)	Mild-Mod TBI (n)	Severe TBI (n)	Cutoff (pg/ml)	Sensitivity (%)	95% CI	Specificity (%)	95% CI	Likelihood ratio	Prevalence (%)	PPV (%)	NPV (%)	Accuracy (%)
IL-6	15	13		> 3.050	77	49.74% to 91.82%	93	70.18% to 99.66%	11.54	46%	90%	82%	86%
IL-6	15		7	> 4.576	100	64.57% to 100.0%	100	79.61% to 100.0%		32%	100%	100%	100%
IL-7	21		9	< 26.24	89	56.50% to 99.43%	57	36.55% to 75.53%	2.074	30%	100%	92%	94%
IL-10	14	14		> 0.3905	79	52.41% to 92.43%	100	78.47% to 100.0%		50%	100%	82%	89%
IL-10	14		9	> 0.3728	100	70.09% to 100.0%	100	78.47% to 100.0%		39%	100%	88%	92%
IL-16	20		9	> 1603	100	70.09% to 100.0%	70	48.10% to 85.45%	3.333	31%	60%	100%	79%
CP IL-8	18	13		< 6842	84.62	57.77% to 97.27%	61.11	38.62% to 79.69%	2.176	42%	61%	85%	71%
GM-CSF	10	10		> 343.7	80	49.02% to 96.45%	70	39.68% to 89.22%	2.667	50%	73%	78%	75%
GM-CSF	10		8	> 346.2	88	52.91% to 99.36%	70	39.68% to 89.22%	2.917	44%	70%	88%	78%
MDC	20	15		> 1553	80	54.81% to 92.95%	55	34.21% to 74.18%	1.778	43%	57%	79%	66%
MCP-1	21		7	> 291.7	71	35.89% to 94.92%	76	54.91% to 89.37%	3	25%	50%	89%	75%
VCAM-1	21	15		< 55944	80	54.81% to 92.95%	57.14	36.55% to 75.53%	1.867	42%	57%	80%	67%
IFN-γ	14	8		< 1.725	88	52.91% to 99.36%	71	45.35% to 88.28%	3.063	36%	64%	91%	77%

CP: Cytokine Panel, GM-CSF: Granulocyte-Macrophage Colony-Stimulating Factor, IL: Interleukin, IFN-γ: Interferon Gamma, MCP: Monocyte Chemoattractant Protein, MDC: Macrophage Derived Chemokine, VCAM: Vascular Cell Adhesion Molecule

Table S4. Sensitivity, Specificity, Positive and Negative predictive values for each Cytokine & Chemokine in predicting Unfavorable Outcomes in a subset of patients with Pediatric Traumatic Brain Injury

GOS-E PEDS	Unfavorable Outcome	Favorable Outcome	**Cutoff (pg/ml)**	**Sensitivity (%)**	**95% CI**	**Specificity (%)**	**95% CI**	**Likelihood ratio**	**Prevalence (%)**	**PPV (%)**	**NPV (%)**	**Accuracy (%)**
IL-5 24HR	5	7	> 4.326	100	56.55% to 100.0%	85.71	48.69% to 99.27%	7	42%	83%	100%	91%
IL-6 0HR	6	10	> 8.135	83.33	43.65% to 99.15%	80	49.02% to 96.45%	4.167	38%	71%	89%	81%
IL-10	8	11	> 2.834	75	40.93% to 95.56%	90.91	62.26% to 99.53%	8.25	42%	86%	83%	84%
IL-16	8	11	> 3998	75	40.93% to 95.56%	90.91	62.26% to 99.53%	8.25	42%	86%	83%	84%
TNF- b	7	6	> 0.2665	71.43	35.89% to 94.92%	83.33	43.65% to 99.15%	4.286	54%	83%	71%	77%
MIP-1α	6	13	< 91.21	100	60.97% to 100.0%	53.85	29.14% to 76.79%	2.167	32%	50%	100%	68%
VEGF	8	13	< 98.62	75	40.93% to 95.56%	84.62	57.77% to 97.27%	4.875	38%	75%	85%	81%

IL: Interleukin, TNF-β: Tumor Necrosis Factor Beta, MIP-1α: Macrophage Inflammatory Protein-1 Alpha, VEGF: Vascular Endothelial Growth Factor
